# How the Learning Path and the Very Structure of a Multifloored Environment Influence Human Spatial Memory

**DOI:** 10.5709/acp-0180-5

**Published:** 2015-12-31

**Authors:** Laurent Dollé, Jacques Droulez, Daniel Bennequin, Alain Berthoz, Guillaume Thibault

**Affiliations:** 1 Perception and Action Physiology Laboratory, Collège de France, Paris, France; 2 Faculty of Mathematics, Team Geometry and Dynamics, Paris Diderot University, Paris, France; 3 Electricité de France R&D, Clamart, France; 4 The Institute for Intelligent Systems and Robotics (ISIR), Pierre and Marie Curie University, Paris, France; 5 Collège de France, Paris, France

**Keywords:** navigation and spatial memory, spatial cognition, representation

## Abstract

Few studies have explored how humans memorize landmarks in complex multifloored buildings. They have observed that participants memorize an environment either by floors or by vertical columns, influenced by the learning path. However, the influence of the building’s actual structure is not yet known. In order to investigate this influence, we conducted an experiment using an object-in-place protocol in a cylindrical building to contrast with previous experiments which used rectilinear environments. Two groups of 15 participants were taken on a tour with a first person perspective through a virtual cylindrical three-floored building. They followed either a route discovering floors one at a time, or a route discovering columns (by simulated lifts across floors). They then underwent a series of trials, in which they viewed a camera movement reproducing either a segment of the learning path (familiar trials), or performing a shortcut relative to the learning trajectory (novel trials). We observed that regardless of the learning path, participants better memorized the building by floors, and only participants who had discovered the building by columns also memorized it by columns. This expands on previous results obtained in a rectilinear building, where the learning path favoured the memory of its horizontal and vertical layout. Taken together, these results suggest that both learning mode and an environment’s structure influence the spatial memory of complex multifloored buildings.

## Introduction

While the brain’s mechanisms for spatial navigation have been extensively studied for planar environments, little is known about human spatial memory in 3D environments like multifloored buildings (Büchner, Hölscher, & Strube, 2007; Christou & Bülthoff, 1999; Hölscher, Meilinger, Vrachliotis, Brösamle, & Knauff, 2006; Jeffery, Jovalekic, Verriotis, & Hayman, 2013; Montello & Pick, 1993; Wilson, Foreman, Stanton, & Duffy, 2004). While Montello and Pick (1993) and Hölscher et al. (2006) suggested that humans recall the positions of landmarks in a building better within than across floors (representation by floors), in Büchner et al. (2007), participants regionalized the building either by floors or by staircases. However the tendency to memorize buildings by floors might stem from the learning mode since in these studies participants mainly explored buildings by floors. This is why Thibault, Pasqualotto, Vidal, Droulez, and Berthoz (2013) studied the acquired memory of landmark locations in a virtual three-floored rectilinear building, in which each floor was a straight corridor divided into three rooms, each one containing a particular object. At learning, participants passively visited the virtual building by following a dedicated learning path, either by floors (named floor learners) or by columns (named column learners), and were instructed to memorize the location of each object. At test, the camera moved from a room containing an object to an adjacent empty room, and participants had to remember and select out of four objects the object that was located in this room. The tests used either a learning path that was familiar to the learner (i.e., by floor for floor learners) or novel (i.e., by column for floor learners). Floor learners obtained better results and shorter reaction times (RTs) in familiar tests (learned paths) than in novel tests, and crucially, so did column learners, which clearly demonstrates the influence of learning path on the acquired memory of landmarks in a building.

However, due to its relative simplicity, the environment’s geometry, with its rectilinear corridors, might have been perceived by some participants as a frontal plane (and not as a building with several 2D floors). This simple structure may have reduced the potential influence of the environment’s structure on the acquired memory. The present study investigated the influence of learning path in a cylindrical building, using the same protocol as Thibault et al. (2013), to contrast the influence of a cylindrical versus a rectilinear structure. We expected the learning paths to have a selective influence depending on the structure learned.

## Method

### Participants

Thirty participants (20 males and 10 females) took part in this study. They are employees of Electricité de France (the main electricity provider in France). None was active in the field of 3D navigation. Their ages ranged from 22 to 54 years (with an average age of 39). All participants had normal or corrected-to-normal vision. This study was approved by the local Ethics Committee. Participants gave their informed consent before starting the experiment.

### Apparatus

**Figure 1.**
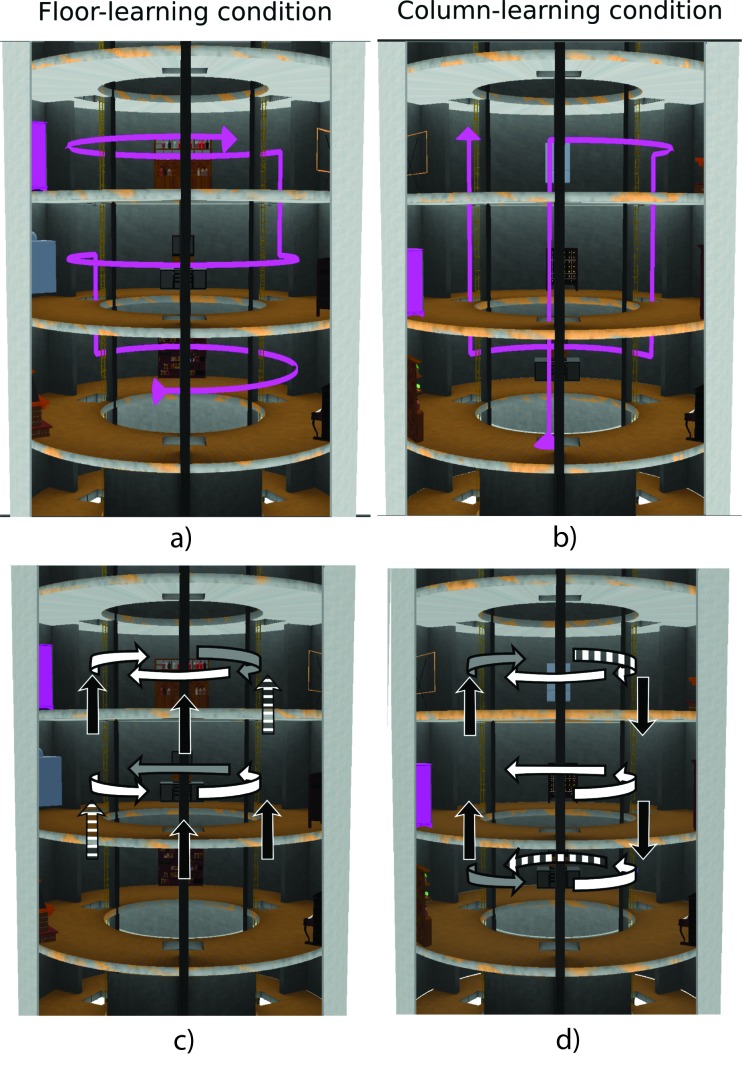
Cutaway of the virtual building. Trajectories (pink ribbons) followed during the learning phase for (a) the Floor-learning group and (b) the Column-learning group. Trial segments for (c) the Floor-learning group and (d) the Column-learning group. Segments are depicted by white arrows for floor trials, black arrows for column trials, grey arrows for closure trials, and white striped arrows for miscategorized trials (not analyzed).

We used a cylindrical virtual building (Figure 1) modelled with SolidWorks and rendered in real-time using Virtools. The environment was displayed on a 30 × 48 cm monitor at a refresh frequency of 60 Hz with a screen resolution of 1,600 × 1,200 pixels and a contrast of 2500:1. The viewers sat about 50 cm from the monitor resulting in a horizontal field of view of 51°. The virtual field of view was set at 73°. The building consisted of three superposed circular corridors (with a 9 m radius), which were themselves divided into three rooms, each of which’s size was equivalent to one third (120°) of the corridor. Two cylindrical walls delimited the corridors: An internal wall (7 m radius), placed in the centre of the building, prevented participants from seeing objects in other rooms, while an external wall prevented participants from seeing outside. As each room included an entrance and an exit, plus ladders to move above and below, it was connected to the next room horizontally and vertically. Stimuli consisted of nine virtual objects: a fireplace, a bar, a children’s writing desk, a bookcase, a boiler, a kitchen unit, a blackboard, a drawer, and a piano. Each of the nine objects stood against the external wall and was placed in the middle of a different room in the virtual building.

### Procedure

This object-in-place experiment consisted of a learning stage followed by a testing stage, with the whole experiment taking about 45 min to complete. Since we adapted the methods of Thibault et al. (2013), unless stated otherwise, our procedure and parameters are equal or equivalent to those chosen in Thibault et al.

#### Learning stage

Participants viewed the virtual building’s corridors. This passive visit led them through the full set of floors and objects twice (number determined in a pilot study). The duration of each exploration was 60 s at a constant speed of 1.35 m/s.

Two learning conditions were tested, each involving 15 different subjects.

a) In the *floor-learning condition*, participants sequentially discovered all the objects on a floor before moving to the floor above (Figure 1a). Contrary to Thibault et al. (2013) the direction of the trajectory was the same throughout all the floors (there was no reversing of the travelling direction between floors).

b) In the *column-learning condition*, they discovered all the objects in a column before moving to the next column (Figure 1b).

Therefore, in the floor-learning condition, the trajectory consisted of six floor segments and two column segments, whereas in the column-learning condition, it consisted of two floor segments and six column segments.

Objects were seen in the same order in each learning trajectory because for each group they were placed in the building accordingly. We have controlled neither the grouping nor the order of objects, since there exists a multiplicity of possible relations (per color, shape, use, name, etc.). However, we counterbalanced these individual choices by recruiting a large number of subjects. The environment was arranged so that objects were always viewed one by one for about 3 s. Participants were informed that each room contained only one object, but they were not told the total number of objects or floors, or that the building was cylindrical. They never saw the building from the outside, nor could they see outside the building. In consequence, it was not possible for them to anchor their orientation on distal cues (see e.g., Knierim & Hamilton, 2011, for a review). They were also instructed to pay attention to the spatial relations between the objects so they could build a “mental representation” of the building.

While the camera moved in facing direction within a floor, vertical transitions were performed sideways, so that the participant could see the ladder scrolls on the left of her/his field of view. A small wall was added in front of the objects so that objects from both floors could not be seen simultaneously during vertical transitions. At the end of the first tour, a text appeared informing the participants they had been relocated to the starting point.

#### Testing stage

**Figure 2.**
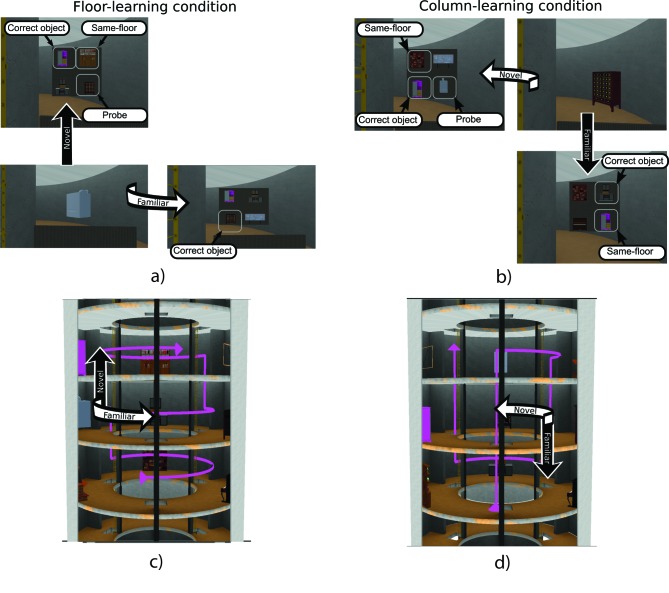
Examples of floor and column segments starting from the same room. (a) Start screen (bottom-left), and arrival screen (top-left and bottom-right) corresponding to the floor-learning condition. (b) Start screen (top-right), and arrival screen (top-left and bottom-right) corresponding to the column-learning condition. (c) Arrows showing trial segments on the floor trajectory. (d) The same for the column trajectory.

Participants underwent trials consisting of a camera movement, called a segment, from one room to an adjacent room (Figure 1c and 1d). In each trial, a fixation cross appeared for about 1 s before the camera view was to a room where the participant could see the corresponding object (Figure 2a and 2b, starting screen).

The camera then moved for about 15 s to an adjacent empty room either on the same floor (floor trial) or in the same column (column trial). Thus, depending on the learning condition, a trial could either replicate a segment of the learning path (familiar segment) or perform a shortcut relative to the learning trajectory (novel segment), as shown in Figure 2c and 2d. Hence, familiarity of the trial is defined as the consistency between the direction of movement on the test segment and the direction of the movement in the same segment during learning. During learning, the cylindrical shape of the building was never explicitly stated: For instance, as shown in Figure 1a, in the third floor, participants had visited the bar/secretary and secretary/blackboard segments, but not the blackboard/bar segment. We called this last segment a “closure” segment, as it would have revealed that the floor was in fact a closed circular path. In order to assess if participants had inferred the cylindrical nature of the building, we also tested two closure segments. Two other segments had been experienced during the learning stage but were at odds with the main direction of the learning condition: They were discarded from the results analysis and called miscategorized segments, since they could not be categorized into any family of segments. Therefore, for each learning condition, out of the 10 trials analyzed, six were horizontal (four floor and two closure segments) and four were vertical (four column segments). In total, two series of 12 segments (the 10 segments analyzed plus the miscategorized ones), ordered in a pseudo-random manner, were run (Figure 1c and 1d). Not all possible segments were run to keep reasonable the whole duration of the experiment.

After 500 ms, four objects appeared (see Figure 2a and 2b, arrival screens) and participants were required to choose the one that was originally located in that room (arrival object, appearing simultaneously with three distractor objects) by pressing the corresponding key among four predefined keys. Participants were encouraged to answer as accurately and as quickly as possible. Response identities and RTs were recorded. Each trial of the floor-learning condition was paired with a trial for the column-learning condition by sharing the same departure object. It was not possible to keep the same arrival objects for novel segments for both conditions, since two objects standing next to each other in the floor-learning condition were not superposed in the column-learning condition.

The trials in which the segment was novel included a particular distractor, the probe distractor, an object adjacent to the departure object along the learning path. Thus, in the floor-learning condition, this object is located in a room on the same floor as the departure object, and in a novel trial (for this condition) it appeared with three other objects in the room of the floor above. Similarly, in the column-learning condition it is located in a room of the same column as the departure object, and in a novel trial it appeared in a room of the column next door. If spatial memory is determined by the sequential order of objects along a learning path, then we should find that probe distractors are chosen more frequently than the other distractors. Another interesting distractor, not present in the experiment of Thibault et al. (2013), was also presented in each trial: In floor- and column-learning conditions a same-floor object was located in a room on the same floor as the arrival object (and differed from the probe distractor). The last distractor was chosen so that among the four objects presented two were from one floor, and the other two were from another floor. In the floor trials of the column-learning condition only, this object was also a same-column distractor, as it was located in the same column as the correct object.

#### Preparation

Participants attended a pre-experiment session (inspired by Huttenlocher & Presson, 1979) designed to assess whether they had any long-term spatial memory impairment. After the pre-experiment, participants underwent a familiarization phase to acquaint themselves with the main experiment. They experienced a simplified version of the main phase in a smaller virtual environment with four objects in four rooms. Participants experienced the same learning condition they had been assigned for the main experiment.

Performance was not recorded, although feedback on the answers given was provided and, in the event of errors, the trial was repeated.

#### Hypothesis

This work reconsidered the three hypotheses studied by Thibault et al. (2013). We argued that if human spatial memory of multifloored environments (here a cylindrical one) is preferentially exploited by floors regardless of the learning mode, then better performance should be observed for floor trials than for column trials. If the use of spatial memory depends on the learning condition, we should observe better performance for familiar segments compared to novel ones. Finally, if learning spatial relations is easier for floor learners, we should observe better performance for floor learners than for column learners in floor trials as well as in column trials.

## Results

All subjects sat through the whole session completely, without experiencing unusual fatigue or nausea. Performance was computed as the rate of correct object selection in the total number of trials. During the testing phase, each participant went through 8 (4 × 2) floor trials, 8 (4 × 2) column trials, 4 (2 × 2) closure, and 4 (2 × 2) misaligned trials, for each type of trial. The two series were pseudo-randomly ordered in order to diminish any precedence effect. The overall mean performance was 57.5% (*SEM* = 11) correct answers, less accurate than in Thibault et al. (2013, 67%). We ran one-sample *t*-tests to compare performance in each elementary condition and found that all but the performance of floor learners in column recognitions, 26%, *t*(15) = 0.1, *p* = .92, were significantly above chance level, *t*(15) > 3.8, *p* < .01, all one-sample *t*-testss are tested with the alternative hypothesis µ > µ_0_ with µ_0_ = 0.25. In floor trials, both floor and column learners yielded performances above the chance level (82% and 52% respectively). Table 1 shows the main performances obtained in this experiment.

**Table 1.** Performances (%) of Learning Groups in the Cylindrical Building

Learning condition	Testing condition	Performance (% of correct answers)
Floor	Floor	82
	Column	26
Column	Floor	56
	Column	69

**Figure 3.**
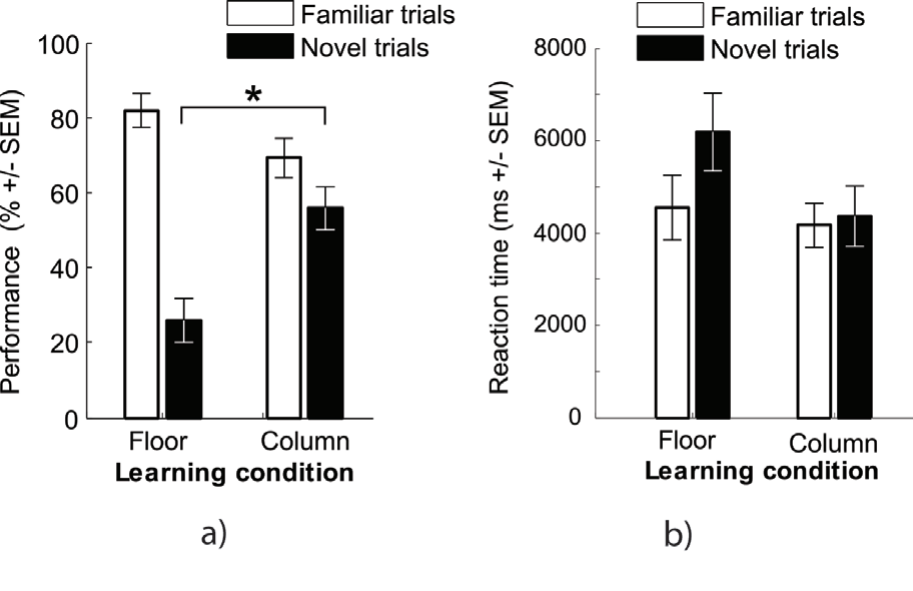
Bar charts of the results. (a) Performance as a function of learning and testing conditions. * the star between black bars emphasizes the significant difference between learning conditions on novel trials. More precisely, column learners outperformed floor learners in novels trials—that is, column recalls for floor learners and floor recalls for column learners (b) Composition of “same-floor plus correct object” responses in novel trials. (c) Reaction time as a function of learning and testing conditions. The error bars represent *SEM*.

The most striking result is thus the poor, chance level performance of floor learners in novel column trials. Performance and RTs were analyzed using a two-way ANOVA between learning and testing conditions (2 × 2 levels, see Table 2), seconded by Kruskal-Wallis tests, more robust to outliers. Firstly, it showed the significant effect that this testing condition has on performance, *F*(1, 56) = 7.93, *p* < .01, also confirmed by a Kruskal-Wallis test, *p* < .05, χ^2^ = 6.44. Indeed, participants performed better in floor trials than in column ones. Secondly, this finding was not significantly dependent on the learning condition, *F*(1, 56) = 1.34, *p* = .25, as confirmed by a Kruskal-Wallis test, *p* = .35, χ^2^ = 0.89. Lastly, we observed an interaction between learning and testing conditions, showing that both groups performed better in familiar than in novel trials, *F*(1, 56) = 21, *p* < .01. However, in novel trials alone, column learners performed better than floor learners: Two Kruskal-Wallis tests conducted for separate learning conditions show that, while floor learners performed better in familiar than in novel trials, *p* < .01, χ^2^ = 13.83, column learners answered equally well in both familiar and novel trials, *p* = .20, χ^2^ = 1.63 (see Figure 3a). Column learners also outperformed floor learners in novel trials, *p* < .01, χ^2^ = 6.65.

**Table 2.** Average Performances of Subjects as a Percentage of Correct Answers

	Testing condition					
Learning condition	Floor	Column	Familiar	Novel	Closure	All trials
Floor learners	82 (10)	26 (11)	82 (10)	26 (11)	68 (8)	54 (11)
Column learners	56 (13)	69 (12)	69 (12)	56 (13)	43 (9)	67 (12)
All subjects	67 (11.5)	47.5 (11.5)	75.5 (11)	39 (12)	55.5 (8.5)	57.5 (11.5)

*Note*. Mean, (*SEM*)

In closure trials, firstly both floor and column learners responded better than chance, as shown by the one-sample *t*-testss, 68% and 43%, *t*(15) > 1.9, *p* < .05, and secondly performances between learning conditions were nearly identical, *F*(1, 56) = 3.76, *p* = .053. Miscategorized trials were also answered better than chance, 58% and 60%, *t*(15) > 5, *p* < .01, and equally well in both floor and column learning conditions, Kruskal-Wallis test, *p* = .84, χ^2^ = 0.04.

In novel trials, distractors were analyzed by comparing their selection rate with the average selection rate. We computed the probability of choosing the probe or the same-floor distractor by chance among the three distractors by following a binomial distribution law *B*(*n*, *p*) where *p* = 1/3 (since it is one of three distractors) and *n* is the number of errors committed (*n* = 45 for floor learners, *n* = 30 for column learners). We only included the first occurrence of each trial (as indicated in the methods all trials are doubled during the testing phase) in order to discard any precedence effect. However, we also ran a binomial test with both occurrences of each trial and we got similar results. Floor learners and column learners respectively selected the probe distractor 8 and 9 times. By comparing these values with a two-tailed binomial test for a confidence interval of 95%, we observed that for floor learners the same floor distractor was selected significantly more frequently than the chance level, 36 selections, *p* < .01, while probe and other distractors were chosen less frequently than by chance by floor learners, respectively, *p* < .01 and *p* < .05. The same method showed that column learners did not choose any distractor more frequently than by chance, including the other distractor, which is also a same column distractor in this very condition, *p* = .80 for probe, *p* = 0.12 for same floor, and *p* = .30 for other/same-column distractors. Binomial tests are summed up in Table 3.

Concerning the RTs, a two-way ANOVA did not show any significant difference between either learning conditions, *F*(1, 56) = 2.64, *p* = .11, or testing conditions, *F*(1, 56) = 1.15, *p* = .29 (see Table 4 and Figure 3c).

**Table 3.** Computed Probability of the Observed Selections of Each Distractor

Learning condition	Probe distractor	Same floor error	Other
Floor learners	*0.17* [0.20; 0.49]	**0.8** [0.20; 0.49]	*0.02* [0.20; 0.49]
Column learners	0.3 [0.17; 0.52]	0.46 [0.17; 0.52]	0.23 [0.17; 0.46]

*Note*. Values enclosed by the confidence interval at 95% following the binomial law of choosing each distractor with a probability of 1/3. Values in bold show computed probability greater than chance level; values in italics show computed probability smaller than chance level.

**Table 4.** Reaction Times for the Different Conditions (s)

Learning condition	Testing condition	
	Floor	Column
Floor	4.5 (0.7)	6.2 (0.8)
Column	4.4 (0.6)	4.2 (0.5)

*Note*. Mean, (*SEM*)

## Discussion

Existing studies on spatial memory in multifloored buildings suggested that people prefer to memorize such environments by floors, and are also influenced by the learning path. Our experiment in a cylindrical multifloored environment expands on these results. We tested floor and column trials in groups that had learned the path either by floors or by columns, and we analyzed how interaction of learning and testing conditions influenced the performance. It significantly showed that the familiarity (floor, resp. column trials for floor, resp. column learners) helped participants to recall connections between objects. However, only column learners could infer the positions of objects in novel-that is, floor trials, while floor learners were unable to infer connections between vertically aligned objects.

Floor learners did not only fail to infer the location of objects across floors: In novel trials, while the correct object was not chosen more often than by chance, the same-floor object was chosen more than twice above chance level. During the learning trajectory, the same-floor object was always seen sooner than the correct one. Therefore, floor learners may have used a “floor-sequence” rule: They chose the first object seen on the right floor during the learning phase.

The poor performance of floor learners may be due to the nature of their learning trajectory which is much curvier, and therefore disorientating, than the column-learning trajectory, having a cumulative turn of 720° compared to 240°. Moreover, no reorientation was possible due to the environment’s circular shape. This has already been pointed out by Kelly, McNamara, Bodenheimer, and Carr (2008) in a spatial updating experiment in planar environments, in which participants walked down a circular path either in a circular, square-shaped, or trapezoidal room. The pointing error increased with the path length in the circular room, but not in the other rooms (see also Shelton & McNamara, 2001, for a similar observation in a judgment of relative direction in circular rooms). In our experiment, such a difference in the disorientation caused by the learning path led floor learners to memorize only the relative location of objects within a floor, and not across floors, while column learners, less disoriented, succeeded in memorizing the relative location of objects both within floors and across floors. The great preference of floor learners in novel trials for same-floor distractors (Table 3, computed probability of being chosen of .80 while it should not have exceeded .40 it was chosen randomly) as well as the good performance of column learners in familiar trials (Table 2, 69% of correct answers) suggest that a primary grouping by floors was at work in both groups.

Our results complete those of the experiment conducted by Thibault et al. (2013) in which the materials and the methods were almost identical, except for the shape of the environment, suggesting that memorization of a cylindrical multifloored building is done by floors. In addition to the familiarity effect, both groups preferred correct and same-floor objects, and thus organized the building by floors. This was not demonstrated in the rectilinear experiment because the building was likely handled like it was a two dimensional frontal environment, but this is in line with neurophysiological studies suggesting that neural bases handling navigation memorize 3D space as a stack of 2D plans. For instance, Hayman, Verriotis, Jovalekic, Fenton, and Jeffery (2011) showed that hippocampal place cells from rats respond more accurately in horizontal dimensions than in vertical dimensions, as if several 2D maps of the environment were stacked. Therefore, a circular path seems to engage a memory of a horizontal plane whereas a rectilinear path may engage a memory of a one-directional space as in Thibault et al. The relatively good performance of both column learners and floor learners in closure trials (respectively 68% and 43% of correct answers, both responded better than chance) suggests that participants were able to take advantage of the circular organization of floors.

However we did not test every segment type, like diagonal ones, in which the participant would be moved simultaneously by floor and by column. Those segments would allow us to better characterize how participants have memorized the environment according to their learning condition. In addition, since our experiment did not involve any actual navigation, we need to extend these findings with a protocol in which participants are free to find their way in buildings exhibiting rectangular or circular 2D floors.

While uncommon, there are cylindrical buildings in the world, such as the “Maison de la Radio” or “Charles de Gaulle terminal 1 airport” in Paris or future Apple headquarter in Cupertino, but also industrial structures such as power plants. For instance, our results can have practical uses for workers who must deal with vertically aligned targets like scaffoldings in offshore platforms or in electric stations: They could benefit from a visit favouring vertical trajectories when discovering these installations.

Our results (see Figure 3) show that, in contrast to a rectilinear environment, a cylindrical environment is better memorized by floors regardless of the learning mode. But only participants who learnt it by columns could also memorize it by columns. Taken together, this work and that of Thibault et al. (2013) show that spatial memory of multifloored environments results from an interplay between the learning mode and the environment’s structure.
